# The Prevalence of Bowel and Bladder Function During Early Childhood: A Population-Based Study

**DOI:** 10.1097/MPG.0000000000003804

**Published:** 2023-04-25

**Authors:** Sanne J. Verkuijl, Monika Trzpis, Paul M.A. Broens

**Affiliations:** From the * Department of Surgery, Division of Pediatric Surgery, University of Groningen, University Medical Center Groningen, Groningen, the Netherlands; the † Department of Surgery, Anorectal Physiology Laboratory, University of Groningen, University Medical Center Groningen, Groningen, the Netherlands.

**Keywords:** constipation, gastrointestinal, incontinence, infant, toddler

## Abstract

Our primary aim was to determine bowel and bladder function in children aged 1 month to 7 years in the general Dutch population. Second, we aimed to identify demographic factors associated with the presence of bowel and bladder dysfunction, and their coexistence.

**Methods:**

For this cross-sectional, population-based study, parents/caregivers of children aged from 1 month to 7 years were asked to complete the Early Pediatric Groningen Defecation and Fecal Continence questionnaire. Different parameters of bowel and bladder function were assessed using validated scoring systems such as the Rome IV criteria.

**Results:**

The mean age of the study population (N = 791) was 3.9 ± 2.2 years. The mean age at which parents/caregivers considered their child fully toilet-trained was 5.1 ± 1.5 years. Prevalence of fecal incontinence among toilet-trained children was 12%. Overall prevalence of constipation was 14%, with a constant probability and severity at all ages. We found significant associations between fecal incontinence and constipation [odds ratio (OR) = 3.88, 95% CI: 2.06–7.30], fecal incontinence and urinary incontinence (OR = 5.26, 95% CI: 2.78–9.98), and constipation and urinary incontinence (OR = 2.06, 95% CI: 1.24–3.42).

**Conclusions:**

Even though most children are fully toilet-trained at 5 years, fecal incontinence is common. Constipation appears to be common in infants, toddlers, and older children. Fecal incontinence and constipation frequently coexist and are often accompanied by urinary incontinence. Increased awareness of bowel and bladder dysfunction in infants, toddlers, and young children is required to prevent these problems from continuing at older ages.

## Graphical abstract

**Figure:**
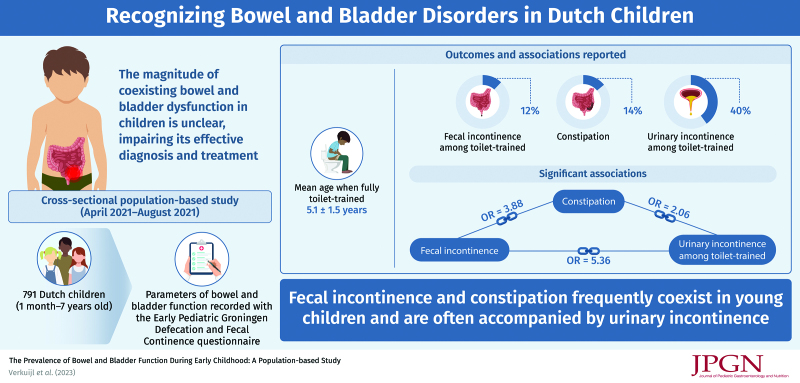

**What Is Known**

- Bowel dysfunction is already prevalent in infants, toddlers, and preschool children.
- However, recognizing bowel dysfunction in young children is difficult, especially because existing studies neither demonstrate the continuity of bowel function from one age to the next nor the magnitude of coexisting bowel and bladder dysfunction.

**What Is New**

- At the age of 5 years most children are toilet-trained.
- Nevertheless, bowel and bladder dysfunction are common and treatment is lacking. Awareness for these conditions in young children is needed to prevent these problems from persisting at older ages.

Bowel and bladder dysfunction are common among children. A recent meta-analysis of population-based studies showed that constipation is prevalent in 10% of children (1). Fecal incontinence is estimated to occur in 1%–4% of all toilet-trained children (2). These bowel complaints impair children’s quality of life and lead to considerable and increasing health care costs (3–5).

Contrary to popular belief, recent studies showed that bowel dysfunction is already prevalent in infants, toddlers, and preschool children (6–11). Existing studies, however, do not demonstrate the continuity of bowel function from one age to the next, but divide their samples into age groups (6–11). The result is lack of insight into the physiological bowel function during the first years of life and this in turn impairs recognizing bowel dysfunction in infants, toddlers, and young children.

According to current literature 3%–47% of constipated children also suffer from urinary incontinence (12). The magnitude of coexisting bowel and bladder dysfunction in infants, toddlers, and young children is unclear. This impairs effective diagnosis and treatment of young children presenting with symptoms of either bowel or bladder dysfunction.

Our primary aim was to determine bowel and bladder function according to age in children aged 1 month to 7 years in the general Dutch population. Second, we aimed to identify demographic factors that were associated with the presence of bowel and bladder dysfunction, and their coexistence.

## METHODS

### Study design

We performed this cross-sectional study between April 2021 and August 2021. Parents/caregivers (hereafter referred to as parents) of children aged 1 month to 7 years were invited to complete the validated Early Pediatric Groningen Defecation and Fecal Continence (EP-DeFeC) questionnaire (13). The EP-DeFeC contains validated scoring systems and criteria regarding bowel and urinary functioning, but also questions regarding demographic characteristics, therapies, and the parents’ classification of their child’s bowel health (13).

Participants were approached by email by an external survey company (Dynata, Rotterdam, the Netherlands). They recruited a randomly selected sample of Dutch-speaking inhabitants from all regions of the Netherlands, whereby the sex and age ratios according to the current population pyramid of the Netherlands, as published by Statistics Netherlands, were taken into account (14). We excluded respondents who reported a physically impossible combination of age, weight, and height according to the Dutch child growth charts (15), and/or invalid answers to open questions. We did not exclude children with congenital diseases or previous surgery. All the questionnaires were completed digitally and anonymously. To avoid missing data, all applicable questions of the digital EP-DeFeC questionnaire had to be completed to submit the questionnaire. Based on the known prevalences of bowel and bladder dysfunction from previous population-based samples, we calculated that a sample size of 800 children would be efficient (see Text, Supplemental Digital Content 1, http://links.lww.com/MPG/D143) (16,17). The study was conducted in compliance with the ethical standards of the Medical Ethical Review Board of University Medical Center Groningen.

### Outcome Measures

We assessed stool consistency, stool frequency, and urinary frequency. We determined stool consistency according to the Bristol Stool Scale, ranging between 1 and 7 with a higher number indicating softer stool (18). Additionally, we examined the prevalence and severity of constipation, fecal incontinence, and urinary incontinence. To define these conditions, we used the validated scoring systems and criteria that are incorporated in the EP-DeFeC questionnaire (13).

We defined constipation according to the Rome IV criteria for neonates/toddlers (0–3 years) and children (≥4 years), depending on the age of the child (2,19). The status of toilet training of the child was defined as “fully toilet-trained,” “is currently being toilet-trained,” or “did not yet start toilet training.” We defined fecal incontinence in fully toilet-trained children as any involuntary loss of stool within the last month. We diagnosed nonretentive fecal incontinence as fecal incontinence without evidence of constipation in children of at least 4 years, according to the Rome IV criteria (2). We defined urinary incontinence according to the definition recommended by the International Children’s Continence Society (20). Additionally, we defined the following 3 subtypes: daytime incontinence, enuresis, or combined daytime incontinence and enuresis (20).

We assessed severity of constipation with the age-adapted Agachan score, ranging from 0 to 30 (21). We assessed severity of fecal incontinence with the Wexner score, ranging from 0 to 20 (22). For both scores a higher score indicates worse functioning. We also used the Pediatric Incontinence and Constipation scores (PICS) for constipation and fecal incontinence, ranging from 0 to 29 and from 0 to 32, respectively (23). Worse functioning is indicated by lower PICS scores. Finally, we asked parents how they would qualify their child’s bowel health and whether they had sought advice regarding bowel dysfunction of their child.

### Definitions of Demographic Characteristics

We gathered the children’s demographic characteristics on sex, weight, and preterm birth from the EP-DeFeC questionnaire (13). We classified the children’s weight as underweight, normal weight, overweight, or obese according to the guidelines of the Dutch association of Pediatrics (24,25). In this way, a weight normal for children younger than 2 years was defined as between −2 SD and +1 SD from the Dutch child growth charts (15,24). A weight lower than −2 SD was defined as underweight (15,25), between +1 SD and +2 SD as overweight, and above +2 SD as obese (15,24). For children aged at least 2 years, we used the standardized body mass index cut-off points (24,25). Preterm birth was defined as birth at fewer than 37 weeks’ gestation, according to the definition of the World Health Organization.

### Statistical Analysis

We expressed continuous variables as mean ± standard deviation (SD) or median and interquartile range (IQR). Categorical variables were shown as number (percentage) and compared using Pearson chi-square tests. We calculated Spearman correlations between age and stool consistency, stool frequency, and urinary frequency. We used cubic spline regression to calculate the probability of constipation, fecal incontinence, and urinary incontinence according to age. We also used cubic spline regression to express the observed and expected constipation and fecal incontinence scores according to age. We used univariable logistic regression analysis to investigate the association between demographic characteristics and constipation, fecal incontinence, and urinary incontinence, and the coexistence of these 3 conditions. We performed all analyses with SPSS software, Version 23.0 (IBM Corp, Armonk, NY) and R Version 3.6.3 (R Foundation of Statistical Computing, Vienna, Austria). We considered *P* values below 0.05 as statistically significant.

## RESULTS

A total of 1021 parents of children aged 1 month to 7 years completed the EP-DeFeC questionnaire. We excluded 215 respondents on account of an invalid combination of the child’s age, weight, and height. Another 15 respondents were excluded on account of illogical answers to open questions. The study population finally comprised 791 respondents. The mean age of the children was 3.9 ± 2.2 years and 55% were boys (see Table, Supplemental Digital Content 2, http://links.lww.com/MPG/D144).

### Stool Consistency, Stool Frequency, and Urinary Frequency

**FIGURE 1.**
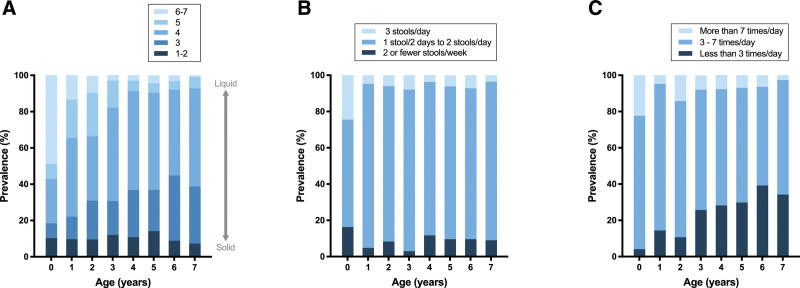
The prevalence of different stool consistencies (A), stool frequencies (B), and urinary frequencies (C) among children from 0 up to 7 years of age.

Mean stool consistency according to the Bristol Stool Scale was 3.8 ± 1.1. Stool consistency was harder in older children (Fig. 1A). Additionally, we found a correlation between children’s age and their stool consistency (rho, −0.249; *P* < 0.001).

Overall, 85% of the children had a stool frequency ranging from 1 stool every 2 days and 2 stools a day. The prevalence of this stool frequency was 59% among children younger than 1 year and varied between 83% and 90% for older children (Fig. 1B). We found no significant correlation between children’s age and their stool frequency (rho, −0.066; *P* = 0.062).

Most children urinated 3–7 times a day (67%). The urinary frequency decreased in older children (Fig. 1C). We found a correlation between the children’s age and urinary frequency (rho, −0.232; *P* < 0.001).

### Toilet Training and Fecal Incontinence

The mean age at which parents considered their child “fully toilet-trained for stool” was 5.1 ± 1.5 years. We found no significant difference between boys and girls (5.0 vs 5.2 years, respectively, *P* = 0.901). The mean age at which parents indicated that their child “was currently being toilet-trained for stool” was 2.7 ± 1.4 years.

**FIGURE 2.**
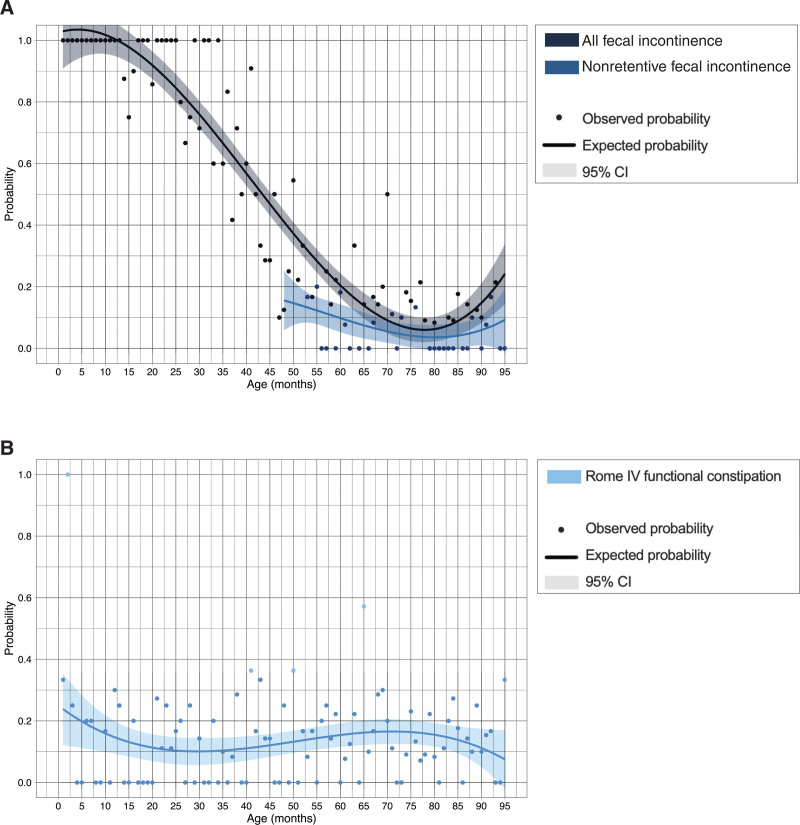
The probability of fecal incontinence (A), nonretentive fecal incontinence (A), and constipation (B) according to age. CI = confidence interval.

The prevalence of fecal incontinence among toilet-trained children was 12% and the prevalence of nonretentive fecal incontinence was 7%. Probability of fecal incontinence decreased in older children and ranged from 1.0 in children younger than 12 months to 0.07 in children aged 77 months (Fig. 2A). Univariable logistic regression analysis confirmed that older children were less likely to suffer from fecal incontinence (OR = 0.98, 95% CI: 0.97–1.00, *P* = 0.013, Table 1).

**TABLE 1.** Prevalence and univariable logistic regression analysis of fecal incontinence, constipation, and urinary incontinence

	Fecal incontinence*			Constipation†			Urinary incontinence‡		
	Prevalence	Univariable logistic regression		Prevalence	Univariable logistic regression		Prevalence	Univariable logistic regression	
Variables	n (%)	OR (95% CI)	*P* value	n (%)	OR (95% CI)	*P* value	n (%)	OR (95% CI)	*P* value
**Demographic characteristics**									
Age, mo	–	0.98 (0.97–1.00)	0.013*	–	1.00 (0.99–1.01)	*0.909*	–	0.97 (0.96–0.98)	< 0.001**
Sex									
Boys	27 (10.3)	Reference		58 (13.4)	Reference		103 (39.8)	Reference	
Girls	34 (13.5)	1.35 (0.79–2.32)	*0.272*	50 (13.9)	1.04 (0.69–1.57)	*0.838*	103 (41.0)	1.05 (0.74–1.50)	*0.771*
Classification of weight									
Normal weight	42 (12.2)	Reference		67 (12.7)	Reference		131 (38.6)	Reference	
Overweight	9 (17.3)	1.51 (0.69–3.31)	*0.309*	13 (15.3)	1.24 (0.65–2.37)	*0.509*	24 (47.1)	1.41 (0.78–2.55)	*0.254*
Obese	7 (10.9)	0.88 (0.38–2.06)	*0.774*	14 (14.7)	1.19 (0.64–2.22)	*0.585*	27 (45.0)	1.30 (0.75–2.26)	*0.354*
Underweight	3 (5.7)	0.43 (0.13–1.45)	*0.173*	14 (16.9)	1.40 (0.74–2.62)	*0.299*	24 (40.0)	1.06 (0.60–1.86)	*0.842*
Preterm birth									
No	55 (11.9)	Reference		97 (13.6)	Reference		180 (39.3)	Reference	
Yes	6 (11.8)	0.99 (0.40–2.42)	*0.977*	11 (14.5)	1.08 (0.55–2.12)	*0.827*	26 (50.0)	1.54 (0.87–2.75)	*0.138*
**Bowel and urinary function**									
Fecal incontinence*									
No				44 (9.7)	Reference		152 (35.2)	Reference	
Yes	N/A	N/A	N/A	18 (29.5)	3.88 (2.06–7.30)	<0.001**	40 (74.1)	5.26 (2.78–9.98)	<0.001**
Constipation									
No	43 (9.5)	Reference					167 (38.0)	Reference	
Yes	18 (29.0)	3.88 (2.06–7.30)	<0.001**	N/A	N/A	N/A	39 (55.7)	2.06 (1.24–3.42)	0.006*
Urinary incontinence‡									
No	14 (4.8)	Reference		31 (10.2)	Reference				
Yes	40 (20.8)	5.26 (2.78–9.98)	<0.001**	39 (18.9)	2.06 (1.24–3.42)	0.006*	N/A	N/A	N/A

CI = confidence interval; OR = odds ratio.

* Only the 513 children who were toilet-trained for stool were included in the analyses.

† All 791 children were included in the analyses.

‡ Only the 510 children who were toilet-trained for urine were included in the analyses.

§Significance of *P* < 0.05,

∥Significance of *P* < 0.005.

**FIGURE 3.**
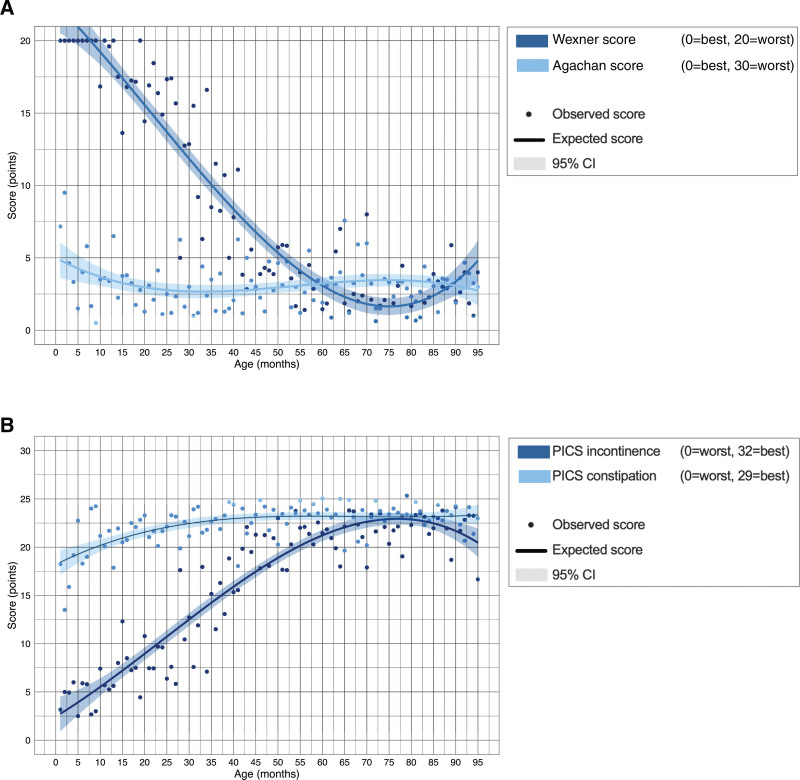
Observed and expected constipation and fecal incontinence severity scores according to age. The Wexner score for severity of fecal continence and the Agachan score for severity of constipation (A), and the PICS incontinence and constipation scores (B). CI = confidence interval; PICS = Pediatric Incontinence and Constipation Score.

The median Wexner score was 0 (IQR 0–5) and the median PICS incontinence score was 23 (IQR 19–26). The Wexner score steeply decreased from 20 in children younger than 12 months to 2 in children of 75 months (Fig. 3A). Likewise, the PICS incontinence score increased from 3 in children aged 1 month to 23 in children aged 75 months (Fig. 3B).

Antidiarrheals were given to 7% of all fecally incontinent children and to 1% of the fecally continent children. Parents of fecally incontinent children did not qualify their child’s bowel health as significantly worse compared to parents of children without bowel disorders (poor to very poor bowel health in 2% vs 1%, *P* = 0.474, see Figure, Supplemental Digital Content 3, http://links.lww.com/MPG/D145). We found that 56% of the parents of fecally incontinent children did not discuss these problems with anyone (see Figure, Supplemental Digital Content 4, http://links.lww.com/MPG/D146).

### Constipation

Overall prevalence of constipation was 14%, with excessive stool retention (75.9%) and painful/hard stools (78.7%) as the 2 most common symptoms. We found that the probability of constipation remained around 0.15 for children at all ages (Fig. 2B). Univariable logistic regression analysis confirmed that constipation was not associated with age (OR = 1.00, 95% CI: 0.99–1.01, *P* = 0.909, Table 1).

Regarding severity of constipation among all the children, the median Agachan score was 2 (IQR 1–4) and the median PICS constipation score was 24 (IQR 21–25). The Agachan score remained around 2.5 at all ages (Fig. 3A). The PICS constipation score increased slightly in older children, ranging from 18 in children aged 1 month to 23 in children of 40 months (Fig. 3B).

Laxatives and enemas were administered to 41% and 9% of the constipated children, respectively, and to 4% and 1% of the non-constipated children. We found that 12% of the parents of constipated children qualified their child’s bowel health as poor to very poor, compared to 1% of the parents of children without bowel disorders (*P* < 0.001, see Figure, Supplemental Digital Content 3, http://links.lww.com/MPG/D145). We found that 28% of the parents of constipated children did not discuss these problems with anyone (see Figure, Supplemental Digital Content 4, http://links.lww.com/MPG/D146).

### Toilet Training and Urinary Incontinence

The mean age at which parents considered their child “fully toilet-trained for urine” was 5.0 ± 1.5 years. The mean age at which parents indicated that their child “was currently being toilet-trained for urine” was 3.0 ± 1.8 years. The prevalence of urinary incontinence among all toilet-trained children was 40%. The majority of these urinary incontinent children suffered from daytime incontinence (56%). Enuresis occurred in 22% and the other 22% suffered from combined daytime incontinence and enuresis.

The probability of urinary incontinence decreased with increasing age from 1.0 in children younger than 15 months to 0.32 in children aged 80 months (see Figure A, Supplemental Digital Content 5, http://links.lww.com/MPG/D147). Univariable logistic regression analysis confirmed that older children were less likely to suffer from urinary incontinence (OR = 0.97, 95% CI: 0.96–0.98, *P* < 0.001, Table 1). Analysis of the subtypes of urinary incontinence showed that while the probability of combined daytime incontinence and enuresis decreased steeply with age, the probability of daytime incontinence and enuresis increased (see Figure B, Supplemental Digital Content 5, http://links.lww.com/MPG/D147).

### Associated Demographic Factors

Neither children’s sex nor preterm birth were associated with fecal incontinence, constipation, or urinary incontinence (Table 1). Likewise, there was no significant association between children’s weight and fecal incontinence, constipation, or urinary incontinence (Table 1).

### Coexistence of Bowel and Bladder Dysfunction

In 29% of children fecal incontinence and constipation coexisted. Univariable regression analysis demonstrated a significant association between fecal incontinence and constipation (OR = 3.88, 95% CI: 2.06–7.30, *P* < 0.001, Table 1). Furthermore, 21% of the urinary incontinent children suffered from coexisting fecal incontinence and 19% from coexisting constipation. We found a significant association between fecal incontinence and urinary incontinence (OR = 5.26, 95% CI: 2.78–9.98, *P* < 0.001) and between constipation and urinary incontinence (OR = 2.06, 95% CI: 1.24–3.42, *P* = 0.006, Table 1).

## DISCUSSION

This population-based study investigated the bowel and bladder function of Dutch infants, toddlers, and children up to 7 years of age. We showed that constipation, fecal incontinence, and urinary incontinence were present in considerable numbers of children and that these conditions frequently coexisted.

Our results also showed that Dutch children usually started toilet training around the age of 2½ years and were fully toilet-trained at the age of 5 years. Compared to studies performed 15 years ago, we found toilet training to be completed at an older age (23,26–28). This may indicate a trend and may be attributed to different potty training routines nowadays (27–29).

No fewer than 12% of toilet-trained children suffered from fecal incontinence, with a steeply decreasing probability and severity at older ages. This curve illustrated the physiological decline in fecal incontinence during the first years of life while acquiring toileting skills. A plateau in probability and severity of fecal incontinence was reached at the age of 5 years, which corresponded with the age at which parents indicated toilet training as completed.

Constipation was prevalent in 14% of the children from 1 month to 7 years, which was comparable with previously reported rates in children of the same age using the Rome III or IV criteria for functional constipation (6–11). Probability and severity of constipation remained constant in children from 1 month to 7 years of age. Furthermore, the overall prevalence of 14% constipation that we found in children up to 7 years old corresponded with previously described prevalence rates among older children using the same criteria (9,10,30). Given the equally high prevalence of constipation from infancy until puberty, it seems that children do not automatically outgrow constipation.

The 2 most common constipation-associated symptoms were excessive stool retention and painful/hard stools, a finding that corroborated with the findings reported by other researchers (6,8,31). This indicates that young, constipated children present with different clinical symptoms than do older children and adults (30,32).

Our results demonstrated that fecal incontinence coexisted with constipation in one-third of the children up to 7 years of age. This was comparable to the 25% coexistence of fecal incontinence and constipation in children aged 8–17 years, but much more than the 15% coexistence in adolescents and 4% coexistence in adults (30,33). This emphasizes the importance of gaining information about obstructive complaints when young children present with fecal incontinence and vice versa. Nevertheless, the majority of the fecally incontinent children suffered from nonretentive fecal incontinence. The proportion of nonretentive fecal incontinence in the current study was higher than previously described (10,34,35), which most likely was a reflection of the younger age of our study population.

Urinary incontinence was present in no fewer than 40% of the children we studied, which was much higher than the prevalence reported in studies including older children (36,37). This corresponds with the steeply decreasing probability of urinary incontinence in older children. A further decline in urinary incontinence with ageing is found in older children (37), although others did not find this relation (36).

On account of our finding that urinary incontinence frequently coexisted with both constipation and fecal incontinence, even more than previously found in older children (12,36), it is important to inquire about lower urinary tract symptoms in young children who present with bowel dysfunction. Further problems may be prevented by timely and adequate diagnosis, as treatment of constipation and/or fecal incontinence often leads to the resolution of urinary incontinence (34).

Similar to previous reports in young children, we did not find a sex predominance for fecal incontinence or constipation (6–8,10). It has been suggested that the frequently reported higher prevalence of functional gastrointestinal disorders in women is related to sex hormones, which are not yet present in “adult concentrations” in young children (6). The fact that we did not find an association between preterm birth and bowel or bladder dysfunction corroborates with previous findings (8,38). Also in line with previous reports, we found no association between excessive bodyweight and constipation (6,8,39). Although a higher prevalence of obesity in constipated children of older ages had been described (40). Likewise, there was no association between underweight and constipation, which had previously been described (6). The number of children with underweight in the respective study sample was limited (6).

Remarkably, only 7% of all fecally incontinent children were treated with antidiarrheals, which are comparable with the treatment rate in older children, but less than that in adults (30). The low treatment rate corroborated with our findings that parents of fecally incontinent children did not qualify their child’s bowel health as poor and that more than half of the parents did not seek help. This may either indicate that parents considered involuntary loss of stool as normal, or it may show the taboo on talking about fecal incontinence. Conversely, almost half of the children who suffered from constipation were treated with laxatives. The majority of parents sought medical advice for their child’s problems regarding constipation, which was more than previously reported (6). Nevertheless, half of the children with constipation and almost all the children with fecal incontinence remained untreated. There is certainly room for improvement here because early treatment appears to be more successful (31,41). For the current treatment of constipation and fecal incontinence in young children, we refer to excellent recent reviews (32,34,42,43).

The strength of our study includes the use of a validated questionnaire and the fact that the study sample comprised a representative cohort of Dutch children in terms of sex and age ratios (14). The most important limitation of the current study is the lack of longitudinal data. Another limitation may be that the questionnaire was completed by the parents, which was inevitable because of the young age of the children we studied. However, this may have influenced the remarkably high prevalence of fecal and urinary incontinence. Furthermore, the parents may have given untruthful answers. We tried to minimize this bias by excluding respondents with physically impossible or illogical answers. Finally, we used the Rome IV criteria for constipation without considering the presence of a large fecal mass in the rectum. In this way, we may have underestimated the prevalence of constipation, although a recent study showed the influence of this criterion to be minimal (44).

## CONCLUSIONS

At the age of 5 years most Dutch children are fully toilet-trained. Nevertheless, fecal incontinence is common even in fully toilet-trained children. Constipation appears to be common as well, with an equally high prevalence in infants, toddlers, and older children. Furthermore, fecal incontinence and constipation frequently coexist and are often accompanied by urinary incontinence, which requires attention. Remarkably, treatment of fecal incontinence and constipation is lacking in most of the affected children. Therefore, awareness is needed of both bowel and bladder dysfunction in infants, toddlers, and young children to prevent these problems from persisting at older ages. This awareness can, for instance, be created during regular developmental check-ups at the child health care center within the first years of life.

## Acknowledgments

The authors thank T. van Wulfften Palthe, PhD for correcting the English manuscript and all respondents for participating.

## Supplementary Material


