# Supplementary material for: The Prevalence of Bowel and Bladder Function During Early Childhood: A Population-Based Study
Source: J Pediatr Gastroenterol Nutr. 2023 Apr 25;77(1):47–54. doi: 10.1097/MPG.0000000000003804 (PMC10259211; doi:10.1097/MPG.0000000000003804)
Supplement: Supplementary file 1 [file mpg-77-47-s001.pdf]

Supplemental Digital Content 1. Sample size calculation

The estimated prevalence from previous studies was around 8% (P = 0.08).(1,2,6-11)

We aimed for a 95% level of confidence (Z = 1.96) and the precision corresponding to the effect size (d = 0.08 / 4 = 0.02).

In this way, the sample size (N) was calculated as:(16,17)

$(Z^2 P(1-P)) / d^2 =$

(1.962 . 0.08(1−0.08)) / 0.0004 =

(3.8416 . 0.0736) / 0.0004 = 706,8544.
