# Supplementary material for: The Prevalence of Bowel and Bladder Function During Early Childhood: A Population-Based Study
Source: J Pediatr Gastroenterol Nutr. 2023 Apr 25;77(1):47–54. doi: 10.1097/MPG.0000000000003804 (PMC10259211; doi:10.1097/MPG.0000000000003804)
Supplement: Supplementary file 2 [file mpg-77-47-s002.pdf]

Supplemental Digital Content 2. Characteristics of the included children

	n (%)
**Overall**	791 (100.0)
**Sex**	
Boys	432 (54.6)
Girls	359 (45.4)
**Age (years)**	
0	49 (6.2)
1	104 (13.1)
2	84 (10.6)
3	101 (12.8)
4	103 (13.0)
5	114 (14.4)
6	125 (15.8)
7	111 (14.0)
**Classification of weight**	
Underweight	83 (10.5)
Normal weight	528 (66.8)
Overweight	85 (10.7)
Obese	95 (12.0)
**Preterm birth**	76 (9.6)
