# Supplementary figures and images for: The Prevalence of Bowel and Bladder Function During Early Childhood: A Population-Based Study
Source: J Pediatr Gastroenterol Nutr. 2023 Apr 25;77(1):47–54. doi: 10.1097/MPG.0000000000003804 (PMC10259211; doi:10.1097/MPG.0000000000003804)

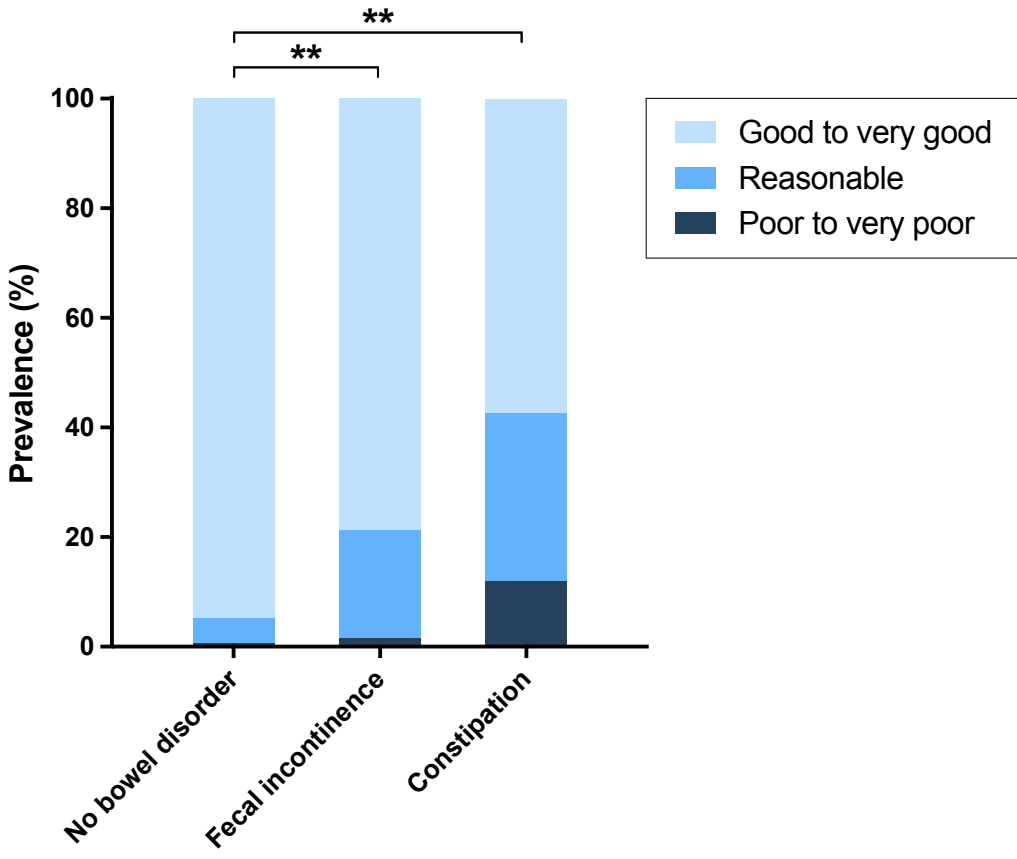

Supplement: Supplementary file 3 [file mpg-77-47-s003.pdf]

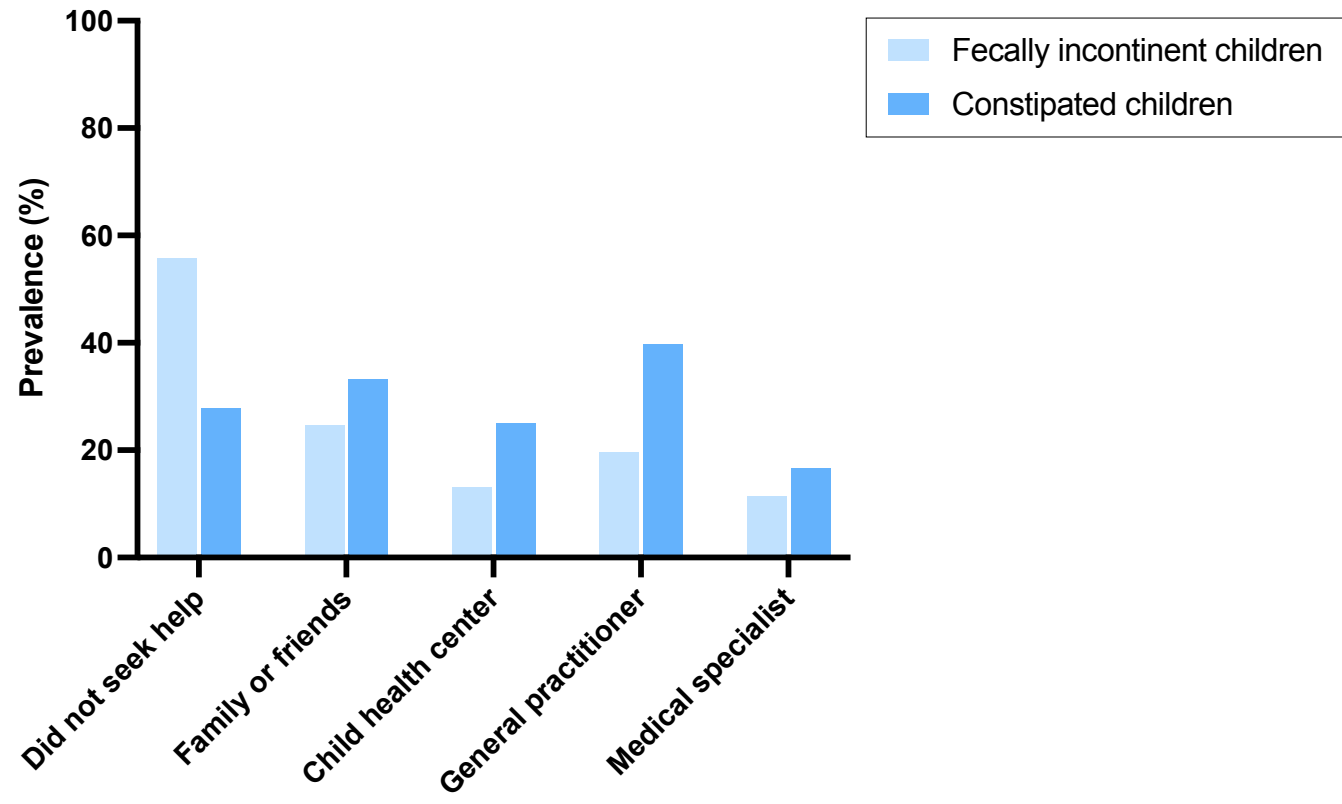

Supplement: Supplementary file 4 [file mpg-77-47-s004.pdf]

**A.**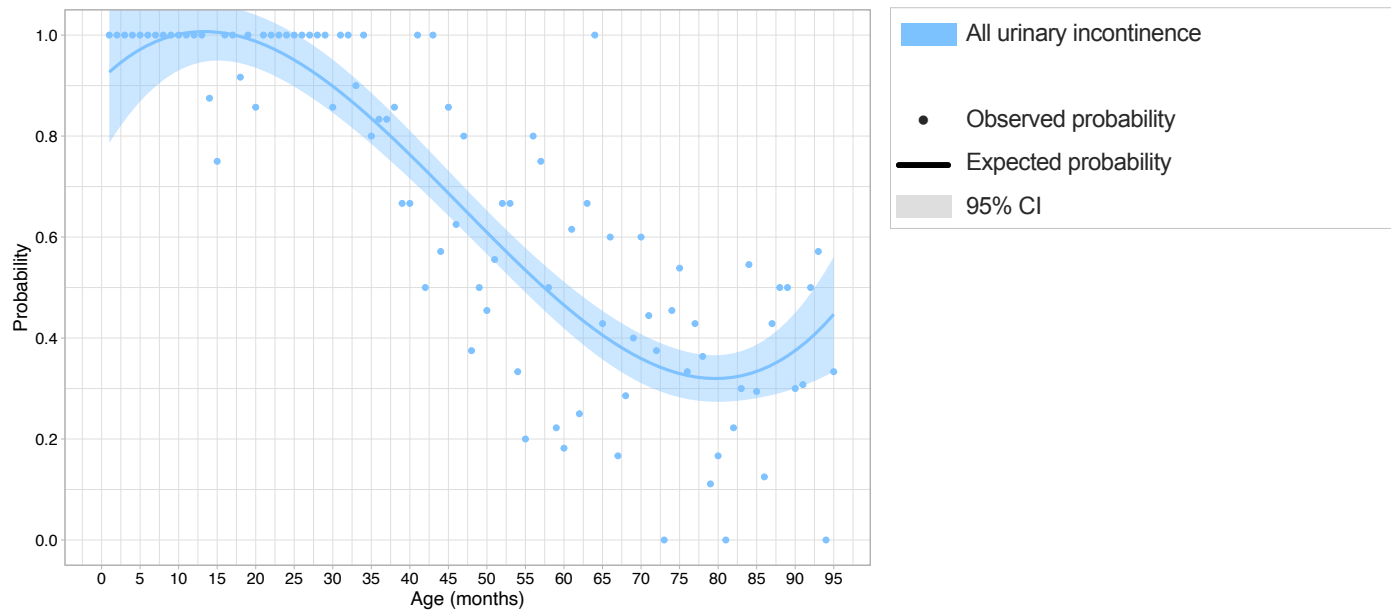**B.**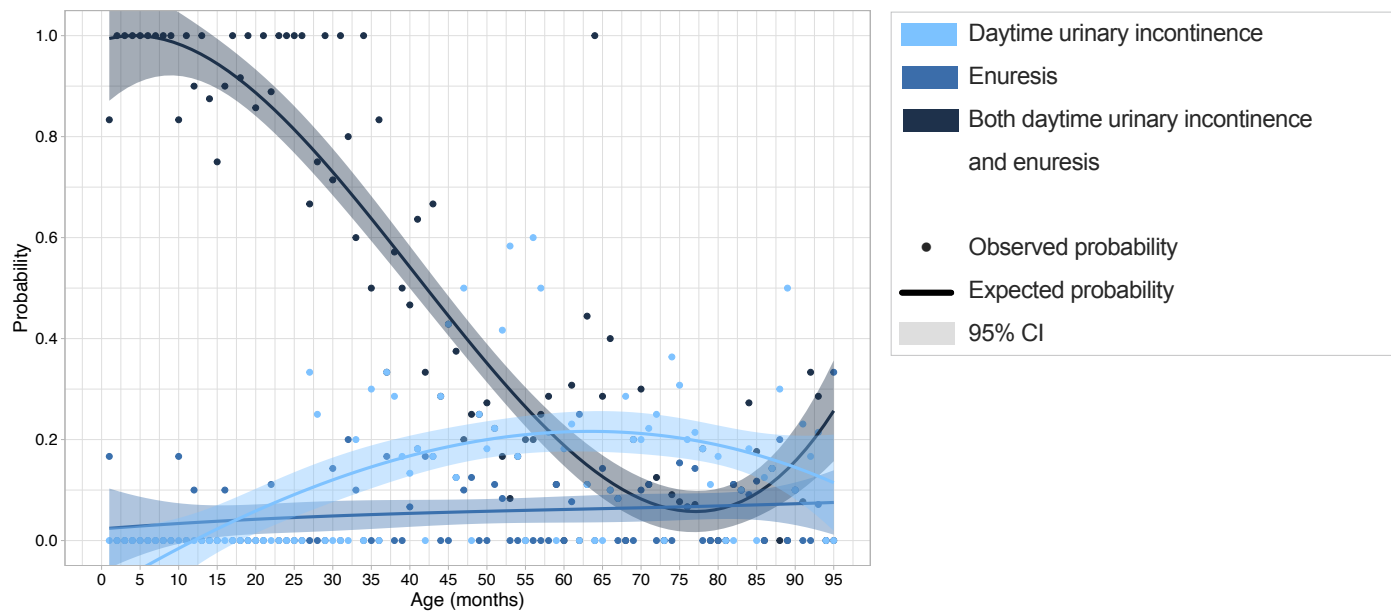

Supplement: Supplementary file 5 [file mpg-77-47-s005.pdf]
